# Compounds from multilayer plastic bags cause reproductive failures in artificial insemination

**DOI:** 10.1038/srep04913

**Published:** 2014-05-09

**Authors:** C. Nerin, J. L. Ubeda, P. Alfaro, Y. Dahmani, M. Aznar, E. Canellas, R. Ausejo

**Affiliations:** 1 Instituto de Investigación en Ingeniería de Aragón (I3A), Universidad de Zaragoza, Campus Rio Ebro, María de Luna 3, 50018 Zaragoza; 2 Department of Research and Development, Magapor SL, Ejea de los Caballeros (Zaragoza), Spain; 3 These authors contributed equally to this work.

## Abstract

High levels of reproductive failure were detected in some Spanish sow farms in the Spring of 2010. Regular returns to estrus and variable reductions in litter size were observed. The problem started suddenly and did not appear to be related to the quality of the ejaculates, disease, alterations of body condition or any other apparent reasons. Subsequent studies determined that the problem was the origin of the plastic bags used for semen storage. Chemical analysis of the suspicious bags identified unexpected compounds such as BADGE, a cyclic lactone and an unknown phthalate that leached into the semen at concentrations of 0.2 to 2.5 mg/L. Spermatozoa preserved in these bags passed all of the routine quality control tests, and no differences were observed between storage in the control and suspicious bags (p > 0.05). In vitro fecundation tests and endocrine profiler panel analysis (EPP) did not show any alterations, whereas the in vivo tests confirmed the described failure. This is the first described relationship between reproductive failure and toxic compounds released from plastic bags.

In intensive pig production systems, 100% of fecundation is performed using artificial insemination. Semen from boars is collected, diluted with an appropriate aqueous solution and finally placed into a high barrier plastic bag until final use. Sperm quality is always checked during processing, and parameters such as motility, morphology, vitality and acrosome integrity are measured before insemination. Sperm is accepted for breeding use only if these parameters achieve the required thresholds.

Reproduction efficiency in swine is high (target > 90% farrowing rate), but some failures (5–7%) are acceptable and the origin can be related to many reasons such as parity, season, stress, poor semen quality, diseases or others^1^. The main failures in reproductive performance are related to non-infectious reproductive problems, such as anestrus, as well as problems related to ovulation, egg production, fertilization, and implantation leading to fetal death or mummification and stillbirth^2^.

From February to April 2010 a dramatic increase in the regular return rate in sow farms was detected. The problem started suddenly in a large swine production system in the South of Spain, with a variable increase in the return rate from 10 to approximately 100%. Regular return to estrus was observed after 17–22 days of insemination with seminal doses (from different boar studs) distributed in plastic bags. In some cases, a reduction in litter size was reported. All internal and external sperm quality controls, such as tests for membrane functionality, abnormal forms, concentration, sperm motility and acrosome status, had produced satisfactory results when the tests were performed during semen processing, transport and farm storage. No changes in health status, feeding strategy or body condition of the sows were observed. Neither disease, season, parity, stress, poor semen nor insemination technique were established as a cause of this problem. After determining the origin of the batch bags with high return rate using a tracking system, the origin of the problem was focused on plastic bags. This hypothesis was reinforced when suspicious bags were removed and replaced by the previous validated batches, and the farms recovered the normal reproduction rates.

In general, semen doses are stored at 17°C in plastic bags for between 24 hours and approximately one week. During this time, compounds present in the plastic bags can leach^3^,^4^,^5^,^6^ into the semen solution and affect the functionality of the spermatozoa. Sperm cells are very sensitive to any compound present in the medium. In fact, boar spermatozoa are frequently used as biosensors for detecting toxic substances^7^.

The effect of xenobiotic compounds such as phthalates on reproduction and fertility has been extensively discussed in the literature^8^,^9^,^10^,^11^, and recently, large lists of endocrine disruptors and reprotoxic compounds have been published^12^,^13^.

In general, the toxicity of individual chemical substances is tested in the laboratory on animals, such as mice, either for oral, dermal or inhalation toxicity, but the toxicity of mixtures of substances has not yet been described. Studies based on direct exposure of potential toxic substances to living cells, such as spermatozoa are scarce. Therefore, the information produced in this study can be of great value.

Plastic bags used for semen storage are commonly manufactured with multilayer materials to achieve high gas barrier properties and to avoid oxidation processes during storage. Multilayer materials contain a combination of different plastics, either coextruded or glued with adhesives. Although the individual materials have been well regulated in some fields such as food packaging (Directive 10/2011/EU^14^, FDA) or pharmaceutical packaging (European and US Pharmacopeia), adhesives are not yet regulated. The general principle of safety and the guarantee that any substance present in the material could endanger consumers health (EU/1935/2004)^15^ is also applied to adhesives, but the controls are not as strict as in other materials. This study provides evidence of the risk we facing, and it emphasizes the importance of adhesive control in packaging^14^,^15^,^16^.

This paper describes a study performed on the packaging materials used for boar semen doses, including the identification of toxic compounds, a migration study and the *in vitro* and *in vivo* tests performed on the semen that was spiked with the chemicals identified as potential reprotoxic compounds in the plastic bags. This study demonstrates the relationship between reproductive failure and compounds released from the multilayer plastic bags used to store semen for artificial insemination. The results obtained are shown and discussed below.

## Results

### Data from affected pig farms

Data collected from all affected farms that suffered from reproductive failure problems are reported. Farms were located in various parts of Spain. Twelve farms were located in the South and Southwest (Andalucía, Murcia, Valencia and Castilla la Mancha), 8 farms were located in the Northwest (Galicia) and 21 farms were located in the North-East (Aragón and Cataluña). The farms housed between 800 and 3000 sows. In all farms, the semen was obtained from different boar studs and was stored in suspicious bags. Boars from the different farms had different genetics, and the reported data were from the same period of time. Only the farms that described the failure problem and shared their data are considered in the study, but no selection of farms was performed. The results shown in table 1 summarizes three periods: the “Before” period: average of reproductive results 5 weeks before the incident; the “During” period: average of 5 weeks during the use of the suspicious bags; and the “After” period: average of reproductive results 5 weeks after the incident. Subsequently, table 1 reports reproductive data from the farms that described the most acute returns that were hypothetically linked to the bags and were considered the basis of this study.

Regular returns were not related to disease, season, parity, stress, poor semen quality or insemination technique. The semen used for breeding was from different boar studs without any detected alteration in spermeograms, and the semen samples met all of the targets defined for standard dose consideration, (total sperm with no more than 75% of total abnormal forms and more than 80% motile sperm). Sow water supply, feed and water used for the semen extender preparation were carefully evaluated and had different origins at the different farms. Microbial contamination of the water or mycotoxins in the feed were carefully ruled out. The health status at all of the farms was evaluated, and none of the following diseases were discovered: leptospirosis, pseudorabies PRV, SMEDI (stillborn pigs, mummified pigs, embryonic death, and infertility), metritis, brucellosis, porcine reproductive and respiratory syndrome (PRRS), porcine circovirus or influenza. The unique common factor to all these farms and boar studs was the use semen bags of the same origin.

The analyzed data clearly showed a significant decrease and variability between farms in terms of fertility as well as total born and alive piglets during the period in which the suspicious plastic bags were used for insemination. Returns observed (table 1) were mostly regular and acutely manifested during the use of the suspicious bags (P < 0.0001 ANOVA test 2 × 2). The percentage of natural abortion was not affected. The fertility decrease varied between farms depending on the duration for which the semen was stored in the bags before being used.

### Analysis and characterization of the plastic bags

**Figure 1:**
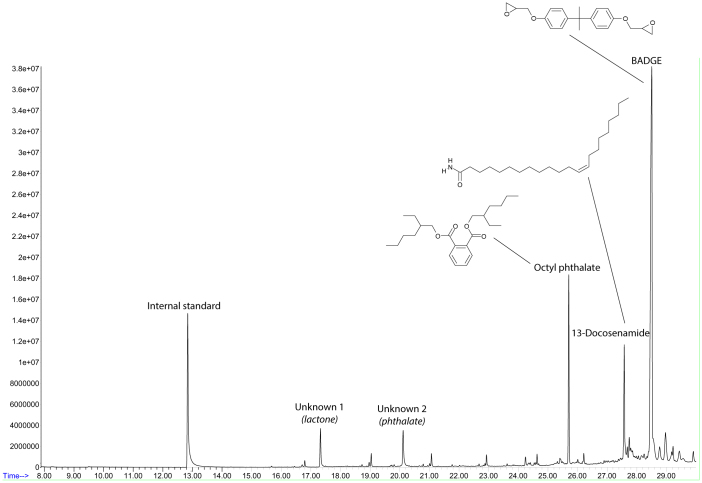
GC-MS Chromatogram of a dichloromethane extract of a suspicious plastic bag.

The analysis of the plastic bags, both the suspicious and control ones, was performed using liquid extraction and gas chromatography- mass spectrometry (GC-MS). These techniques allowed the analysis of volatile and semi-volatile compounds with a high sensitivity. The comparison between chromatograms of suspicious bags and control bags showed 5 peaks only present in suspicious samples (Figure 1). A solid phase microextraction (SPME) coupled with GC-MS analysis of the plastic bags was also performed to increase the sensitivity of the detection of the most volatile compounds, although any additional compounds were detected.

Three peaks were identified using the NIST (National Institute of Standards and Technology) mass spectra library, as octyl phthalate, 13-docosenamide (erucamide) and BADGE (bisphenol A diglycidyl ether). However, the NIST and Wiley mass spectra libraries did not provide any information regarding the other two peaks: unknown 1 and unknown 2. Nevertheless, their mass spectra patterns were linked to a lactone and a phthalate structure, respectively. Both had been previously detected in several multilayer food packaging materials during the development of the MIGRESIVES project^3^,^16^, but none of them were specifically identified. In the MIGRESIVES samples, the individual substrates and the adhesives used for building the multilayer materials were available, and the origin of these compounds was found to be the adhesive. For this reason, it was expected that the adhesive used in the manufacture of the multilayer material was the origin of the compounds.

A solution of adhesives each containing the unknowns 1 and 2 peaks was analyzed using UPLC-MS-Q-TOF (ultrahigh performance liquid chromatography-mass spectrometry-quadrupole-time of flight). This analysis allowed the accurate mass determinations of both the precursor ion and the product ions, providing information about the fragmentation patterns. This technique, together with MassFragment® software from Waters and the Chemspider [www.chemspider.com] and Scifinder [www.scifinder.com] chemical databases facilitated the correct identification of the unknown compounds. Unknown 1 was identified as 1,4-trioxacyclotridecane-8,13-dione, a cyclic lactone. The identification was confirmed by injection of the pure compound isolated from an adhesive. This lactone was also identified in other food packaging materials, and in all cases, the polyurethane adhesive was the source^17^,^18^. Its structure suggests that it is most likely derived from adipic acid. It is a neoformed compound from the ingredients used in the adhesive formula. There are no available toxicity studies for this compound, but Cramer classifies it as Class III, high toxicity, based on its chemical structure^19^.

Two possible candidates were investigated for unknown 2, both with a phthalate structure: diethylene glycol cyclic phthalate (candidate 1) and 4,6-diethoxy-2-benzofuran-1,3-dione (candidate 2). Because no pure standards of these candidates were commercially available, the structure of this unknown was confirmed by NMR. For this purpose, this compound was first isolated using an HPLC system from a pure adhesive sample and identified as the main source of unknown 2. The results obtained were consistent with the proposed candidates, but no clear identification was achieved. Based on the toxicity pattern later described, the hypothesis of the cyclic phthalate has been considered. These are again non-intentionally added substances (NIAS) that are neoformed from the ingredients of the PU adhesive formula and were completely unknown until now.

**Figure 2:**
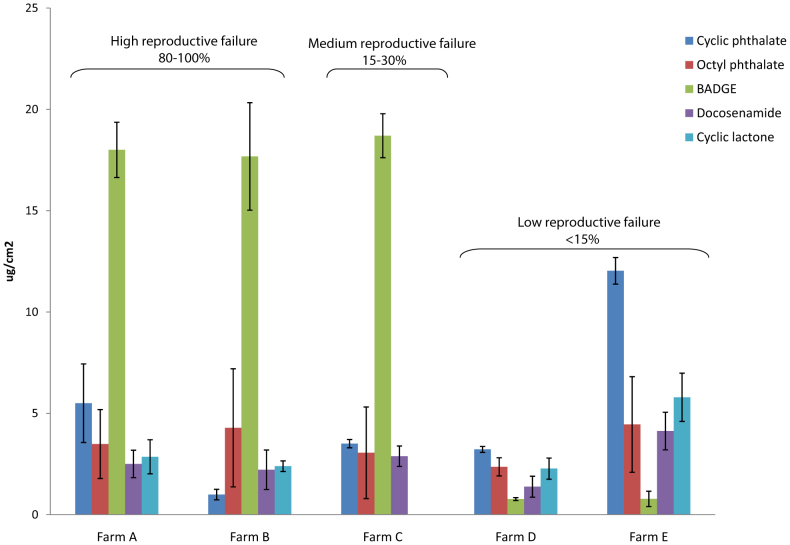
Concentration of compounds found in the different plastic bags sets and relative incidence of reproductive failure observed.

Once the compounds present in the suspicious bags were identified, they were quantified in more than 500 plastic bags used in different farms and with a different percentage of sow returns. Figure 2 shows the concentration of the compounds in the different plastic bag sets and the observed relative incidence of reproductive failure.

### Migration study

**Figure 3:**
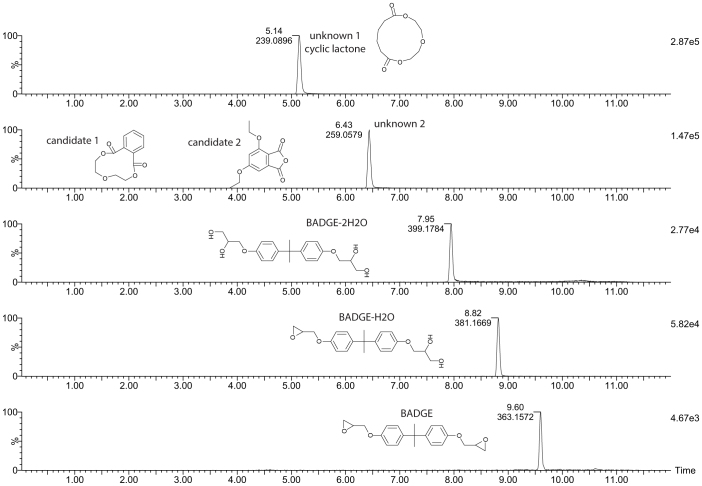
LC-MS Chromatogram of a migration solution (water) from suspicious bags.

A migration study was performed to confirm the transference of these compounds to the semen solution. This study also provided information on the presence of other possible compounds such as material additives or non-intentionally added substances (NIAS). For the migration experiments, plastic bags were filled with Milli-Q water or a long-extender solution and stored at 37°C for 48 hours. The migration solutions were then analyzed using UPLC-QTOF-MS. The high concentration of some long-extender components made the quantification of the studied compounds more difficult and for this reason, an aqueous solution was used for the migration study. Figure 3 shows the migrating compounds from the suspicious bags. BADGE and two diol derivatives, BADGE-H_2_O and BADGE-2H_2_O, which were recognized as toxic compounds^20^ were identified. These compounds have been previously described when BADGE was in contact with aqueous systems, and especially in acidic media because it decomposes and produces the diol-derivatives^21^,^22^, which are at least as toxic as the original BADGE. For this reason, in food-contact materials, the total migration of BADGE derivatives is limited^14^. In addition, the cyclic lactone and cyclic phthalate previously detected by GC-MS were observed. The ratio between the concentration of lactone and BADGE derivatives in the suspicious plastic bags (<0.2) was different from the ratio in the migration solution (~6), as migration of the cyclic lactone was much higher than that of BADGE, and BADGE reacted with water and formed BADGE diols.

**Figure 4:**
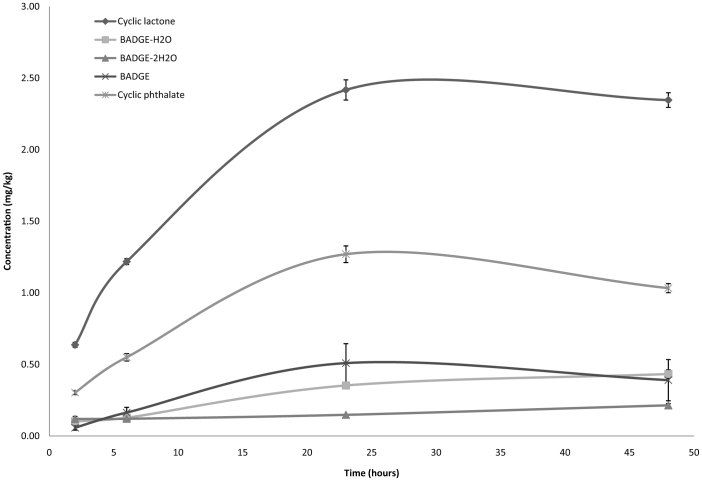
Evolution of migrants concentration.

The evolution of the migrants identified in the aqueous solution exposed to the suspicious plastic bags was studied. The results showed that after 24 hours, the maximum migration values were reached in aqueous solution (Figure 4). The highest migration value was determined for cyclic lactone, 2.50 (±0.05) mg/L, and a value of 1.50 (±0.03) mg/L was determined for cyclic phthalate. BADGE migration increased as well as neoformed BADGE compounds, BADGE-H_2_O and BADGE-2H_2_O, reaching concentrations of 0.40 (±0.14), 0.40 (±0.03) and 0.20 (±0.01) mg/L, respectively. However, after 30 hours, the BADGE concentration slightly decreased while the concentration of diols increased. This result was expected because these diols are generated from BADGE.

Although the compounds responsible for the toxicity were determined, it is important to determine the step at which toxicity develops. For this reason, additional tests were performed.

### *In vitro* tests

#### In vitro seminal analysis

Spermatozoa were tested in direct contact with the potential toxic compounds. Sperm quality parameters were tested and no abnormal results were observed (Table 2) in any of the groups, P (storage) and P (storage × treatment) were above 0.05 based on the ANOVA test.

#### In vitro fecundation test

The aim of this test was to highlight the action of identified toxic compounds in suspicious semen bags based on the oocyte penetration process by spermatozoa. For this reason, the tests were performed in different groups of sperm: sperm stored in suspicious bags, sperm stored in control bags and sperm stored in glass vials and spiked with the different toxic compounds.

The results did not show any significant difference in terms of the penetration rate nor in the penetrated sperm per oocyte between the groups (Table 3) (P > 0.05 ANOVA test).

#### Endocrine profiling panel (EPP) tests

As no detectable alteration of standard semen quality control indicators or of the in vitro fecundation test was observed, a new experiment EPP test was proposed to rule out any alteration of the three endocrine receptors (estrogen alpha, androgen and progesterone) and to identify any potential compound-induced endocrine disruption using simultaneous detection and analysis of multiplexed cellular targets and properties. This test allowed the toxicity of a compound to living cells to be determined. Therefore, it provided the possibility of testing the toxicity of individual compounds as well as their combination in aqueous solution.

All positive controls worked and produced positive responses as expected. However, no cellular toxicity was observed in the samples spiked with the toxic compounds, even at the highest concentrations, neither in the migration samples from suspicious bags, using both %Viability and Toxicity Index measurements (Supplementary Figure 5A and 5B). No response from any receptor measurements (Foci Count, Area, Intensity for all assays, Receptor degradation for ER, and Nuclear Translocation for AR and PR) was found in the samples.

After analyzing all of the EPP results for the test samples and examining the images (Supplementary Figure 5A and 5B), the treatment conditions that were investigated with the test compounds (stored at room temp and heated to 37°C for 24 hours) were negative for receptor activation and toxicity for both AR and ERa. This was the case for both agonist and antagonist assays for ERa because all of the test compound results did not differ from the vehicle controls. It should be noted that positive controls were included for each experiment, and their response was as expected.

#### In vivo fecundation tests

To confirm the reproductive failure, an *in vivo* fecundation test was performed using sperm diluted with Vitasem® extender and spiked with a mixture of toxic compounds (2.5 mg/L of cyclic lactone, 1.5 mg/L of cyclic phthalate, 0.4 mg/L BADGE, 0.4 mg/L of BADGE-H2O and 0.2 mg/L of BADGE-2H_2_O). Breeding of sows was performed in two groups of 50 sows (2 replicates of 25 for each case: sperm with toxic compounds and sperm control). A significant reduction in total born was observed when spiked sperm was used instead of control sperm. The fertility rate decreased from 84 to 58%, and total born decreased from 231 to 70 (P < 0.0001). The results obtained confirmed the previous findings.

### Post-mortem uterus flushing of inseminated sows

Post-mortem uterus flushing was performed on sows inseminated with both sperm stored in control and in suspicious bags.

Post-mortem collection of embryos demonstrated the reproduction failure that was previously observed (Table 4). A significant variation in the fertility rate and total collected embryos by flushing was clearly observed between the control group and the sows inseminated with sperm stored in the suspicious bags (P = 0.0027 and P < 0.0001, respectively, ANOVA test).

## Discussion

A group of pig farms suffered a significant and variable decrease in fertility, total born and alive piglets over the same period of time. The only feature in common was the origin of the plastic bags used for semen storage, and for this reason, the hypothesis that the plastic bags were the cause of the reproductive failure was proposed.

Five different compounds were detected in the plastic bags; some of them, such as cyclic lactone or cyclic phthalate, had been previously found in adhesives used for the manufacture of multilayer food packaging materials, as mentioned above^17^. For this reason, adhesives were identified as the origin of these compounds.

The analysis of the plastic bags, which were recovered from the farms that had reported reproductive failures at different percentages, implicated BADGE, a derivative of Bisphenol A, classified by the International Agency for Research on Cancer [www.IARC.org] as group 3 (Not classifiable as to its carcinogenicity to humans), as the main compound responsible for the failures. In addition, a synergistic effect could be attributed to the presence of cyclic lactone, whereas the cyclic phthalate did not affect the final results, as shown in Fig. 2. Bags from farm C for example, contained a high concentration of BADGE but did not contain cyclic lactone, and the reproductive failure was lower than that in farms A and B, which had a similar concentration of BADGE but higher levels of cyclic lactone. BADGE present in the bags partially reacted with the aqueous solutions to form BADGE-H2O and BADGE-2H2O and therefore, these derivative compounds were also responsible for reproductive failures.

The concentration of toxic compounds in semen depends on the migration processes. It is worth mentioning that the total concentration of BADGE compounds and their derivatives found in migration tests complied with the European Regulation 10/2011/EU, as it was lower than the established specific migration limit of 9 mg/Kg food. The toxicity, when the toxic compounds are in direct contact with sperm, is therefore very different from the toxicity of compounds when they are present in food; this difference can be explained by the differences in the mechanisms of action.

Migration processes depend on a variety of factors, such as contact time, temperature or initial content of the compounds in the plastic material. For this reason, the migration of toxic compounds will be higher in the semen doses stored for longer periods in plastic bags or in bags with a higher content of migrants.

The semen storage time depends on factors such as the weeks of insemination service, farm location and the frequency of semen distribution. The farms located at long distances or those that receive semen less than 5 days a week, require longer storage periods for the semen doses. In addition, multilayer plastics from the same batch can contain either different amounts or different types of adhesive in their manufacture. This situation explains why different concentrations of toxic compounds were found even in bags from the same batch. Methods based only on the study of damage to the sperm cells are commonly used in reproductive toxicity studies. However, the most dangerous reprotoxicity occurs, as it is reported in this case, when no cell damage can be detected by routine analysis and only the clinical signs at the farm indicate problems. This paper demonstrates that reproductive failures can occur even when internal quality control tests were not able to detect any alteration of quality parameters and the endocrine profiling panel provided negative results.

To date, no information that correlates the compounds present in plastic semen bags with the reproductive failure in sows is available. This paper is the first description of the interaction between different compounds and reproductive failure. Unfortunately, it was not possible to prevent the reproductive failure before this study, because the relationship between unexpected unknown substances in the multilayer plastic bags and the reproductive failure was completely unknown and was thus, undetectable. This fact highlights the importance of traceability of packaging materials and the study of migration phenomena, which can strongly affect consumer health. Neoformed compounds and non-intentionally added substances (NIAS) present in packaging materials should be taken into account to assess the safety of the use of multilayer plastics, either for animal reproduction or for human uses. Further investigations are needed to try to determine the proper quality controls in relation to cytotoxicity and/or reprotoxicity that could help animals and human reproductive companies avoid this type of risk for existing or future new discovered substances.

These results also highlight the importance of evaluating the toxicity of the compounds not only individually but also in combination.

ToxInsight™ Endocrine Profiler Panel (EPP) Assays were performed to identify potential compound-induced endocrine disruption by simultaneous detection and analysis of multiplexed cellular targets and properties by testing the estrogen receptor alpha (ERa), the androgen receptor (AR) and the progesterone receptor. Endocrine disruptors are compounds that alter steroid activity thereby perturbing endocrine system functionality. Environmental endocrine disruptors have been linked to numerous adverse health effects and reproductive problems in both humans and wildlife^23^,^24^.

The steroid hormones play a central role in the reproductive events associated with pregnancy establishment and maintenance^25^. In parallel, androgens/androgen receptor (AR) signaling is involved in the development of the female reproductive organs and their functions, such as ovarian folliculogenesis, embryonic implantation, and uterine and breast development^26^.

Nevertheless, no alteration or cell toxicity was observed in this test, which suggests that other systems should be investigated in the future to elucidate the mechanism of action of toxic compounds on the post fertilization processes.

The findings lead us to the hypothesis that damage was likely caused in a later stage of fecundation, probably during implantation of the blastocyst or in the early multiplication and development stages, and that most likely, these toxic compounds did not affect the capacitation, acrosome reaction, sperm recognition and penetration of oocyte zona pellucida. The mechanism of action that caused the reproductive failure and the regular return to heat should be investigated in the future. In parallel, further studies are needed to provide new efficient tools and/or tests to be implemented during batch evaluation of all materials related to human and animal *in vitro* reproduction techniques.

From this study, relevant conclusions can be obtained: a) several toxic compounds such as BADGE and a cyclic lactone (1,4-trioxacyclotridecane-8,13-dione) were found in plastic bags used for preservation of boar seminal doses; b) the origin of these toxic compounds was the adhesive used to manufacture the multilayer plastic bag; c) migration to the semen solution of the compounds identified in the bags and of the neoformed compounds (BADGE-H_2_O and BADGE-2H_2_O), which were produced by direct contact of the plastic bags with the aqueous solutions, was detected and measured; d) a synergistic reprotoxicity effect developed between BADGE, their derivatives and the cyclic lactone (1,4-trioxacyclotridecane-8,13-dione was found; e) no alteration in the semen parameters was observed during semen processing, analysis assessment, preservation quality controls nor in the *in vitro* penetration test; f) *in vivo* experiments confirmed that these compounds found in the aqueous phase were the main cause of reproductive failure in sows; and g) in this case, reprotoxicity did not affect semen quality, partially affected the fecundation step, but most likely affected the implantation, blastocyst or later stages. This is the first time that a reprotoxic effect in pig production has been demonstrated *in vivo* by direct exposure of spermatozoa to potential toxic compounds.

## Methods

### Plastic bags samples

Different batches of plastic bags for semen storage were bought from an international supplier and provided by the company that participated in this study. Plastic bags were classified as “suspicious bags”, those related to reproduction problems, and “control bags”, those without any influence on reproductive results.

For the characterization of the plastic bags, more than 500 suspicious bags were analyzed. They were new and empty and were recovered from 5 different farms (A, B, C, D and E), which reported different rates of reproductive failure.

### Reagents

Dichloromethane (chromatography grade) was from Scharlab. Water and methanol (LC-MS grade) were supplied by Baker (Deventer, Holland).

Commercial standard bisphenol A diglycidyl ether (BADGE), bisphenol A (2,3-dihydroxypropyl)glycidyl ether (BADGE-H2O), bisphenol A bis(2,3-dihydroxypropyl)glycidyl ether (BADGE-2H2O) and *p-tert*-butylphenol were purchased from Sigma-Aldrich (St. Louis, Mo, USA). The compounds 1,4-trioxacyclotridecane-8,13-dione (cyclic lactone) and the cyclic phthalate were isolated from adhesives by liquid chromatography, and they were used as standards for quantification.

### GC-MS analysis

GC-MS was performed on a 6890 series gas chromatography coupled to a 5973 MS from Agilent (Palo Alto, CA, USA). The separation was achieved with a 30 m × 0.25 mm I.D DB-5MS column (J&W Scientific, Folsom, CA, USA) coated with 5% phenyl-95% dimethylpolysiloxane (film thickness 0.25 μm).

The oven temperature was programmed as follows: 40°C (holding time 2 min) to 100°C at 15°C/min to 220°C keeping this condition for 2 min; 1 μl were injected in splitless mode. Helium was the carrier gas (1 mL/min), and the inlet temperature was 270°C.

### LC-MS analysis

Liquid chromatography was performed using a Waters Acquity Ultra Performance LC system (Waters Corporation, Milford MA, USA) composed of a binary solvent delivery manager, a thermostated autosampler and column oven compartment. The chromatographic separation was performed using an Acquity UPLC BEH C18 1.7 μm 2.1 × 100 mm column, which was maintained at 30°C. Mobile phase flow was 0.3 mL/min and the gradient program is shown in table 5. Detection and identification were carried out using a Xevo-QTOF-MS equipped with an electrospray ionization (ESI) source (Waters Corporation, Milford MA, USA). QTOF-MS was operated in positive ionization mode. Detection parameters are shown in table 5. Quantification was performed in a triple quadrupole TQ from Waters, equipped also with an ESI source in positive ionization mode. Acquisition was performed in SIR (single ion recording) mode and protonated molecular ions [MH+] were monitored.

### Methodology for plastic bags analysis by SPME-GC-MS

Cut-offs of plastic bags (2.5 × 2.5 cm) were placed in 20 mL glass vials and the headspace was analyzed by SPME. A PDMS fiber was used. MS detection was performed in SCAN mode in a mass range of 45–450 uma. Five replicates of each type of samples (suspicious bags and control bags) were analyzed, and the chromatograms were compared.

### Methodology for plastic bag analysis by liquid extraction and GC-MS

Cut-offs of plastic bags of 0.25 g (<2 cm) were extracted with 5 mL of dichloromethane and 100 μL of internal standard (*p-tert*-butylphenol, 500 μg/g) were added. The extraction was performed using the ultrasound equipment at 40°C during 30 minutes. Afterwards, the extract was gently evaporated to dryness under a nitrogen current and redissolved with 1 mL of dichloromethane.

### Migration study of the plastic bags

Migration studies were performed on the suspicious bags. Bags were filled with 20 mL of Milli-Q water and also with 80 mL of a long-term extender (VitasemLD, Magapor SL) and placed in a horizontal position so that the liquid was in contact with the entire surface of the bags. Each experiment was performed in triplicate. Bags were kept in the oven at 38°C for 48 hours and then, the aqueous phase was analyzed by UPLC-MS(QTOF) and UPLC-MS(TQ).

### Study of migration evolution

Six suspicious and 3 control bags were filled with 45 mL of Milli-Q water and kept at 38°C. An aliquot of 0.5 mL was taken from the bags at various times: 2 h, 6 h, 23 h and 48 h; then, the samples were analyzed by UPLC-MS(TQ). The evolution of lactone, phthalate, BADGE, BADGE-H2Oand BADGE-2H2O was studied. For this purpose, calibration curves of the studied compounds were built and analyzed. Because there was no phthalate standard it was quantified with the lactone standard.

#### Boar semen samples

Semen samples were obtained from 5 boars of proven fertility that produced one heterospermic sample with appropriate sperm motility, morphology and concentration. Animals were currently in use at Cinse SL AI station (Zaragoza, Spain), and their semen production data were obtained and analyzed using Gesipor 2.0 software (Magapor SL). Boars were held in individual pens with surface areas of 6 m^2^ and concrete slatted floor and sides that allowed physical and visual contact between animals, consistent with European Directive 91/630/CEE. Boars were exposed to both, natural and artificial light for a total of 14 h/day. They were fed with 2.5–3 kg/day of standard feed, and they had access to fresh water *ad libitum*.

#### Semen handling and assessment

One ejaculate was collected weekly from each of the 5 selected boars over 7 weeks because a higher collection rhythm could result in detrimental effects on sperm quality^27^. Samples were collected into pre-warmed insulating collection flasks, within which was a 450 mL plastic container with a filter and 100 mL of pre-warmed (37°C) extender to minimize thermal shock and sperm agglutination^28^. Every semen collection was carried out by the same designated person using double-gloved hands to minimize bacterial contamination. Only the ‘rich’ fraction of the ejaculate was collected, and the sample was analyzed by the same technician on each occasion. Semen was assessed in line with established protocols at the AI center. Sperm motility analysis was performed using a commercial computer-assisted sperm analysis system (CASA; ISAS Proiser, Spain). This system was based on the analysis of 25 consecutive digitalized photographic images obtained from a single field at magnification × 100 in a negative phase contrast field with heated stage (Nikon Eclipse 50i; 10 × 0.30 PLAN objective lens). All materials used were pre-warmed to 36–37°C, and two motility parameters were assessed: percentage of motile spermatozoa (PMS) in at least three 100× microscopic fields; and quality of spermatozoa movement (QSM) in three 400× fields. Sperm morphology and vitality and hypoosmotic Swelling Test (HOST) were evaluated following eosin–nigrosin staining^29^ of at least 200 spermatozoa at 1000× magnification. Acrosomal integrity was evaluated as previously described^30^. The semen concentration was calculated using a Bürker chamber at a 1/100 dilution (sperm/0.3% formol saline solution). Minimal values assumed were as follows: a PMS of 80; a QSM of 4; 80% normal, alive spermatozoa; 90% of spermatozoa with intact acrosomes; and 50% of spermatozoa reacting to the HOST. Semen was diluted in long-term extender (Vitasem LD, Magapor SL) to produce doses of 30 million sperm/ml and placed in plastic bags of 90 mL volume. All 5 ejaculates formed a heterospermic semen batch that was used for spermatotoxicity and in vitro fecundation test.

### Methodology for spiking semen with toxic compounds

Sperm from several boars was diluted in long-term extender (Vitasem®, Magapor SL) and introduced into control bags; then half of the doses were spiked with the compounds previously identified and at the same concentration levels found in the migration tests (2.5 mg/L of cyclic lactone, 1.5 mg/L of cyclic phthalate, 0.4 mg/L BADGE, 0.4 mg/L of BADGE-H2O and 0.2 mg/L of BADGE-2H_2_O). Pure standards of these compounds were prepared in aqueous solutions, and several replicates were tested. For the cyclic lactone and the cyclic phthalate compounds, without an available commercial standard, the isolate pure compounds were used. The same replicates of seminal doses without toxic compounds were used as a control group.

### *In vitro* fertilization test

The test consists of checking the sperm penetration rate of immature oocytes from prepubertal gilts following the described procedures^31^. Briefly, oocytes were obtained from ovaries of prepubertal gilts at a local slaughterhouse and transported to the laboratory at 37 C within 1 h in DPBSm medium. A fraction of 100 μL of semen was washed three times by centrifugation at 1900 rpm for 3 min in mDPBS. The pellet was resuspended in fertilization medium, and sperm concentration was adjusted to produce a final optimum ratio of 2000:1 spermatozoa/oocyte. Sperm and oocytes were co incubated at 39 C under 5%CO_2_ in air for 16 h–18 h. Past 18 h, presumptive zygotes were removed from fertilization medium, washed three times in DPBSm medium to eliminate the excess of spermatozoa and cumulus, then fixed for at least 72 h in fixing solution slides (1:3; acetic acid: ethanol) and then stained with Lacmoid (1%). The penetration rate (presence of female pronucleus and one of more swollen sperm heads and/or male pronuclei with its corresponding head and two polar bodies/total inseminated), monospermy rates (oocytes containing only one sperm head or male pronuclei/total penetrated) and efficiency of fertilization (monospermic oocytes/total inseminated) were measured.

An in vitro penetration test was performed to determine the possible effect of toxic compounds on fertilization. Semen doses from heterospermic doses as described in the previous paragraph were split into different groups and spiked with toxic compounds as follows:

Group 1: Sperm stored in control plastic bags.

Group 2: Sperm stored in suspicious plastic bags.

Group 3: Sperm stored in glass vials without any contact with plastics.

Group 4: Sperm stored in glass vials without any contact with plastics and spiked with 0.4 mg/L BADGE, 0.4 mg/L of BADGE-H2O and 0.2 mg/L of BADGE-2H_2_O).

Group 5: Sperm stored in glass vials without any contact with plastics and spiked with 2.5 mg/L of cyclic lactone.

Group 6: Sperm stored in glass vials without any contact with plastics and spiked with 1.5 mg/L of cyclic phthalate.

Group 7: Sperm stored in glass vials without any contact with plastics and spiked with 2.5 mg/L of cyclic lactone, 1.5 mg/L of cyclic phthalate, 0.4 mg/L BADGE, 0.4 mg/L of BADGE-H2O and 0.2 mg/L of BADGE-2H_2_O.

The concentration of the migrating compounds from the suspicious plastic bags was determined. For each group, 3 plates of 25 oocytes were tested during 7 consecutive weeks. Not all oocytes included in the test could be analyzed due to various factors, such as rupture or alteration during the washing processes.

### Analysis of endocrine profiler panel (EPP)

Endocrine Profiler Panel (EPP) monitors the following: i) cell toxicity through changes in nuclear morphology and count and ii) receptor activity by measuring GFP (green fluorescent protein) intensity and location. EPP involves two assays. Each has a U2OS cell line expressing the estrogen receptor (ERa) tagged to GFP and the androgen receptor (AR) tagged to GFP. A similar assay using the progesterone receptor (PR) was also developed and tested. Experiments were performed and analyzed using EPP dose range instructions. The assays were optimized using samples diluted in cell media. Replacement of the media with distilled water slightly altered the expected response for controls (in all three assays).

Estrogen receptor α, androgen receptor and progesterone receptor assays were performed according to the manufacturers' instructions (ToxInsight EPP, Thermo Fisher Scientific) with the following modifications. Control compounds were diluted in distilled water prior to addition to the cells. Aliquots of 100 μl of aqueous solutions (samples or controls) were added directly to the cells (already in 100 μl of culture media); therefore, the maximum concentration of the samples was 0.5×. Sample concentrations that showed less than 80% viability compared to vehicle control were flagged as toxic. To be flagged as a positive for receptor responses, treatment doses had to exhibit >20% normalized response compared to a positive control.

The suspicious plastic bags were filled with Milli-Q water, and after one week, the water was analyzed by EPP. Several standard solutions containing the individual standards of the compounds discovered in the migration studies as well as the combination of the standards were analyzed.

All three cell lines (estrogen receptor α, androgen receptor, and progesterone receptor) followed identical procedures described in detail in the TFS EPP dose range assay. The cell lines express GFP-tagged fusion proteins that monitor receptor activation by the formation of nuclear foci following stimulation. Secondary outputs of receptor degradation (ERα) and cytoplasm to nucleus translocation (AR and PR) are also monitored to determine the mechanism of action for compound stimulation. Cell viability and toxicity is monitored in each well by counting the nuclei in each image. For the biological protocol, 6000 cells per well were plated into duplicate 96-well microplates and incubated for 16–20 hours at 37°C. Controls and samples (duplicate wells in a 7-point 1:10 dilution series with a maximum concentration of 0.5×) were added to the wells and incubated for 20–24 hours at 37°C. The cells were fixed with 4% formaldehyde, washed with PBS and stained with Hoechst. Images were acquired using optimized Cellomics Compartmental Analysis (V4) protocols on a TFS ToxInsight CHPS or Nxt platform with a 20× objective. Data analysis was performed using the EPP Excel template analysis and confirmed by manual inspection of the images. Samples were repeated in at least 2 separate experiments, and the results were combined for the final analysis.

The following samples were prepared and tested: a) 0.5% dimethylsulfoxide (DMSO) as the blank control; b) the following positive controls where the final concentrations were made in 0.5% DMSO in distilled water: Era = 17b-estradiol at 250 nM; AR = dihydrotestosterone (DHT) at 250 nM; PR = progesterone at 500 nM; c) one aqueous solution in glass vials containing only the cyclic lactone (LG), and this solution was 1 mM; d) one aqueous solution in glass vials containing BADGE, BADGE-H2O, BADGE-2H20 (B) at 1 mM each; and e) one aqueous solution containing the 4 compounds (B + LG diluted) at the concentrations detected in the migration study at levels between 1 and 10 μM.

In addition, 2 different suspicious plastic bags filled with deionized water incubated at 37°C for 24 hours were also analyzed for EPP. Control water was tested as a blank sample.

### *In vivo* tests in sows

*In vivo* tests were performed in sows inseminated with semen doses spiked with toxic compounds.

All procedures were carried out under Project License PI 35/13 approved by the in-house Ethics Committee for Animal Experiments from the University of Zaragoza. The care and use of animals were performed accordingly with the Spanish Policy for Animal Protection RD53/2013, which meets the European Union Directive 2010/63 on the protection of animals used for experimental and other scientific purposes.

For in vitro experimentation, semen was obtained from a commercial boar stud called Cinse SL, as described in a previous paragraph, located near the farm and laboratories where the analysis were carried out. Oocytes were extracted from ovaries recovered from routine slaughtered sows.

The study of sow insemination to confirm the reproductive failure with isolated compounds was carried out under field conditions in Valporgen S.L, a commercial pig farm in Spain, due to the closeness to the other places involved in the study and because of the health status of the farm, with an A4 health status level, which was free of porcine reproductive and respiratory syndrome virus (PRRS).

Semen diluted in long-term extender (Vitasem LD, Magapor SL) was split in two groups: 1) control group, and 2) case group, which was spiked with combinations of the above-mentioned toxic compounds. Semen doses were filled in ‘blister’ bags of 90 mL and stored at 15–17°C until insemination of the sows. Receiving sows were composed of a homogeneous population of 100 weaned hybrid multiparous, Large White x Landrace. The sows were placed in a mating – control unit and supplied with a standard lactation diet in liquid feeding system. Estrus detection was performed once a day in the morning; it started on the second day after weaning (weaning day considered as day 0) and it was consistently performed by the same experienced operator every day. Sows were inseminated with a post-cervical insemination system in two or three services (until the end of estrus) every 24 h depending on the individual sow requirements. From two consecutive weaned sows batches (n = 50 sows) with a weaning to service interval (WSI) of 4, 5 or 6 days, half of them were randomly attributed to the control group and half to the case group [(25 + 25) × 2].

### Post-mortem flushing and collection of embryos

This experiment was performed under the same conditions as described in the previous paragraph but using sows in heat that were going to be culled because their age (more than 7 litters). Forty-five sows from 4 farms that used the control semen plastic bags for insemination, and 42 sows from 4 farms using the suspicious plastic bags were subjected to post-mortem embryos flushing.

Sows were sacrificed in the slaughterhouse 7 days after mating with semen doses that were stored in the control bags or the suspicious bags, and the genital tracts were immediately recovered. At this time, embryos were in the blastocyst stage and were located in the uterine horn. Extraction of the embryos was carried out using a Foley catheter placed in the extremity of the horn. At the beginning of each horn, 50–150 mL of phosphate buffer (BPS) was injected with a syringe and in the other horn's extremity, a Foley Catheter was introduced through a small incision and fixed by inflation of the balloon. Buffer with embryos was collected in a falcon tube at the catheter end by the slight application of pressure with the hands on the uterine horns. After sedimentation, drops of pellet with embryos were placed in Petri plates, examined under a stereomicroscope and counted.

### Statistical analyses

The results of the quantitative parameters are represented as the mean ± SD (standard deviation) and analyzed using ANOVA 2 × 2 and Fisher PLSD test, checking the normality of each group according to the Kolmogorov Smirnov test. Normal or categorical parameters were represented as percentages and were analyzed using the Post-hoc and Chi-squared methods. Differences among the parameters were considered significant when P < 0.05. All the statistic tests were performed using StatView 5.0 software, SAS Institute Inc.

## Author Contributions

C.N., P.A., M.A. and E.C. performed the GC-MS and LC-MS analysis of the plastic bags, the identification of unknown compounds, migration studies and quantification of migrants. J.L.U., Y.D. and R.A. performed the semen assessment and analysis, in vitro tests with sperm, in vitro fecundation and in vivo fecundation tests.

## Supplementary Material

Supplementary Information Supplementary Figure 5A and 5B

## Figures and Tables

**Table 1:** Percentage of farrowing rate in sows and litter size of piglets in 41 farms before, during and after using suspicious plastic semen bags periode. (P value: P of ANOVA/Fisher PLSD Test. Values are represented as mean ± standard deviation)

	Breeded sows, (N)	Farms, (N)	Fertility (%)	Regular return (%)	Total born/sow	Total born alive (N)	Born alive/sow	Abortions (%)
**Before**	15685	41	85.9 ± 3.0	10.8 ± 3.0	11.3 ± 1.1	133953	10.7 ± 1.1	1.9 ± 0.2
**During**	13970	41	56.3 ± 14.5	39.6 ± 14.5	9.4 ± 1.5	55928	8.6 ± 1.6	1.3 ± 0.2
**After**	12655	41	82.4 ± 2.9	12.4 ± 2.9	11.8 ± 0.9	105802	11.1 ± 0.9	1.3 ± 0.2
**P value**			<0.0001	<0.0001	<0.0001	<0.0001	<0.0001	0.43

**Table 2:** Quality parameters in semen spiked with the toxic compounds after 1, 3 and 6 days of storage. T-Mot: Total Motility, Viab: Viability, Shost: Hypoosmotic Swelling test, ORT: Osmotic Resistance Test. P value: P of ANOVA/Fisher PLSD Test. Values are represented as mean ± standard deviation

Storage days	Treatment	T-Mot	Viab	Shost	ORT
**1**	Control	85.0 ± 7.1	91.0 ± 4.2	69.0 ± 1.4	84.0 ± 0.0
	Toxic compounds	82.5 ± 6.4	89.5 ± 5.4	73.0 ± 3.9	84.5 ± 3
**3**	Control	72.5 ± 3.5	84.0 ± 2.8	67.5 ± 4.9	77.0 ± 1.4
	Toxic compounds	72.5 ± 2.8	82.0 ± 2.8	64.5 ± 3.4	77.0 ± 3.8
**6**	Control	72.5 ± 3.5	72.0 ± 2.2	69.0 ± 4.2	80.5 ± 0.7
	Toxic compounds	71.2 ± 2.5	71.0 ± 8.6	69.7 ± 1.2	79.0 ± 4.7
**P values**	P (Storage)	0.0015	0.005	0.07	0.01
	P(Treatment)	0.58	0.59	0.72	0.84
	P(Storage × treatment)	0.9	0.98	0.26	0.88

**Table 3:** Penetration test in different groups of sperm samples

	N (total oocytes)	Penetration rate (%)	Penetrated sperm/oocytes (%)
**Group 1**	462	75.54 ± 3.3	2.05 ± 0.47
**Group 2**	467	76.97 ± 4.3	1.97 ± 0.39
**Group 3**	287	75.20 ± 2.5	1.70 ± 0.50
**Group 4**	468	73.28 ± 2.3	1.99 ± 0.17
**Group 5**	359	72.65 ± 2.3	1.90 ± 0.48
**Group 6**	392	74.68 ± 1.7	2.15 ± 0.20
**Group 7**	362	78.17 ± 1.7	2.07 ± 0.13
**P -value**	-	**0.115**	**0.847**

(P value: P of ANOVA test. Values are represented as mean ± standard deviation).

Group 1: Sperm stored in control plastic bags.

Group 2: Sperm stored in suspicious plastic bags.

Group 3: Sperm stored in glass vials without any contact with plastics.

Group 4: Sperm stored in glass vials without contact with plastics and spiked with BADGE and the two BADGE-diols.

Group 5: Sperm stored in glass vials without contact with plastics and spiked with cyclic lactone.

Group 6: Sperm stored in glass vial without contact with plastics and spiked with cyclic phthalate.

Group 7: Sperm stored in glass vial without contact with plastics and spiked with BADGE,BADGE-diols, cyclic lactone and cyclic phthalate.

In each case the concentration of the compounds was the same as that obtained in the migration from the suspicious plastic bags into water.2.5 mg/L of cyclic lactone, 1.5 mg/L of phthalate, 0.4 mg/L BADGE, 0.4 mg/L of BADGE-H2O and 0.2 mg/L of BADGE-2H_2_O.

**Table 4:** Flushing and embryos post-mortem collection from breeded sows with semen stored in suspicious and control plastic bags (Average of 4 farms using suspicious bags and 4 farms using control bags)

	Breeded sows (N)	Fertility (%)	Total Embryos (N)
**Control bags**	45	82.0 ± 5.1	231.0 ± 13.7
**Suspicious bags**	42	55.0 ± 9.9	70.0 ± 20.5
**P-Value**		P = 0.0027	P < 0.0001

(P value: P of ANOVA test. Values are represented as mean ± standard deviation).

**Table 5:** Instrument parameters for the UPLC- MS analyses

UPLC parameters		
Flowrate	0.3 mL min^−1^	
Column temperature	30°C	
Injection volume	5 μL	
Gradient time table		
Time (min)	% Methanol	% Water
0	5	95
5	100	0
8	5	95
10	5	95
Electrospray MS parameters		
Desolvation gas flow	600 L h^−1^	
Cone gas flow	60 L h^−1^	
Desolvation gas temperature	450°C	
Source temperature	150°C	
Capillary voltage	3 kV	
