# Supplementary material for: Compounds from multilayer plastic bags cause reproductive failures in artificial insemination
Source: Sci Rep. 2014 May 9;4:4913. doi: 10.1038/srep04913 (PMC4015085; doi:10.1038/srep04913)
Supplement: Supplementary Information — Supplementary Figure 5A and 5B [file srep04913-s1.pdf]

COMPOUNDS FROM MULTILAYER PLASTIC BAGS CAUSE REPRODUCTIVE FAILURES IN ARTIFICIAL INSEMINATION.

C. Nerin[1]§, J.L.Ubeda[2*], P. Alfaro[1*], Y. Dahmani[2*], M. Aznar[1*], E. Canellas[1*] and R. Ausejo[2*]

Supplementary Figure 5A and 5B: EPPS supplementary data

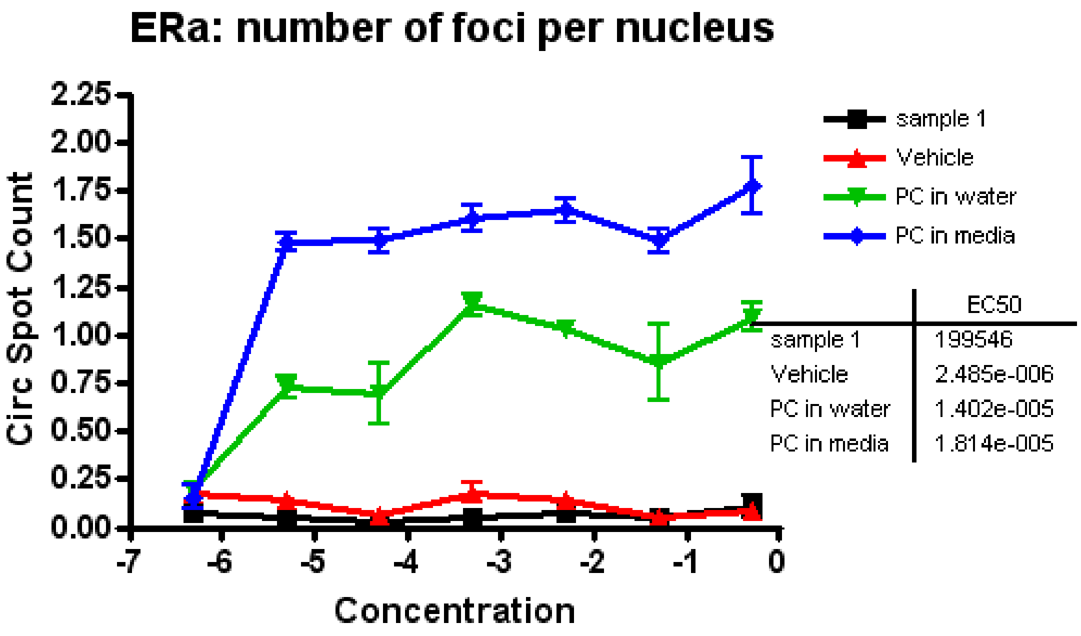
ERa: number of foci per nucleus
Circ Spot Count
2.25
2.00
1.75
1.50
1.25
1.00
0.75
0.50
0.25
0.00
-7
-6
-5
-4
-3
-2
-1
0
Concentration
sample 1
Vehicle
PC in water
PC in media
EC50
sample 1 199546
Vehicle 2.485e-006
PC in water 1.402e-005
PC in media 1.814e-005

#

#

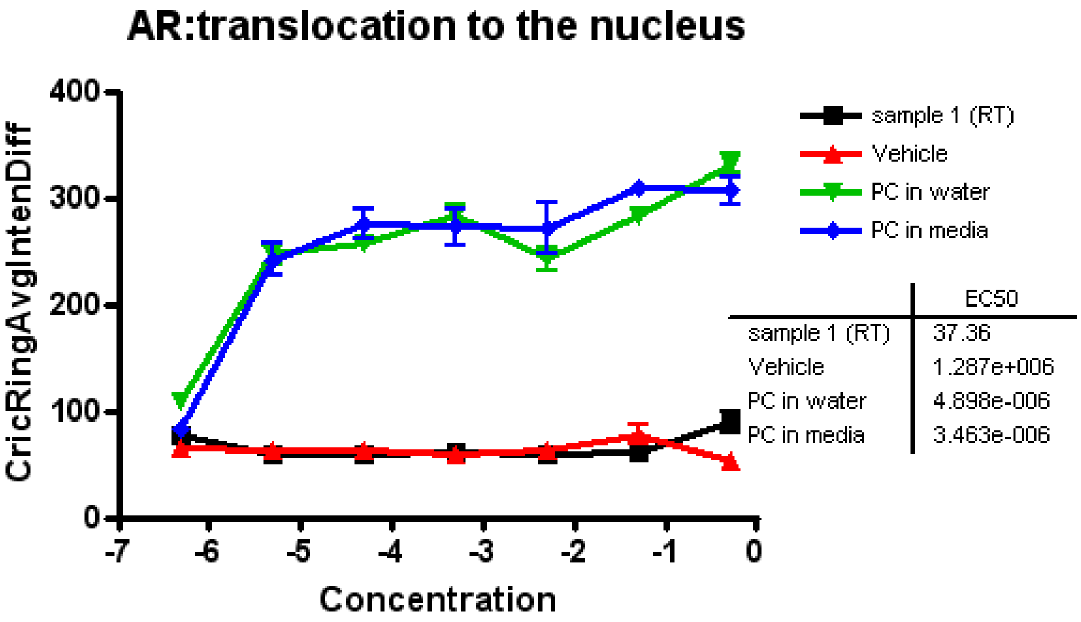
AR:translocation to the nucleus
CricRingAvgIntenDiff
400
300
200
100
0
-7
-6
-5
-4
-3
-2
-1
0
Concentration
sample 1 (RT)
Vehicle
PC in water
PC in media
EC50
sample 1 (RT) 37.36
Vehicle 1.287e+006
PC in water 4.898e-006
PC in media 3.463e-006

#

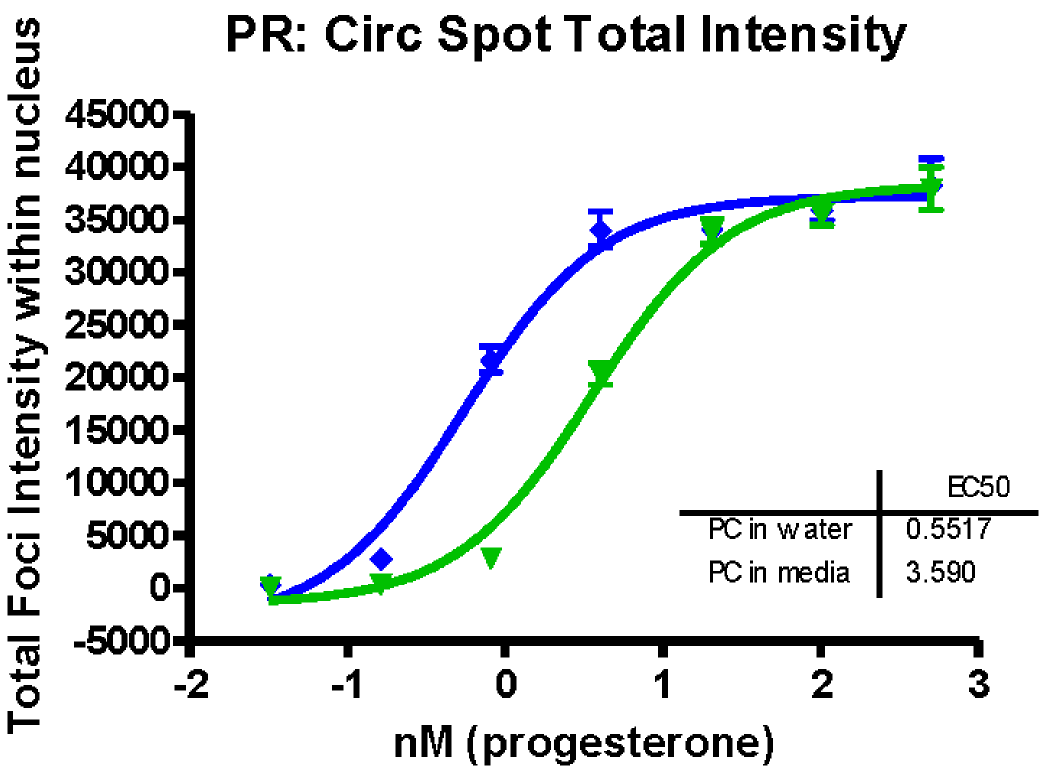

#

#

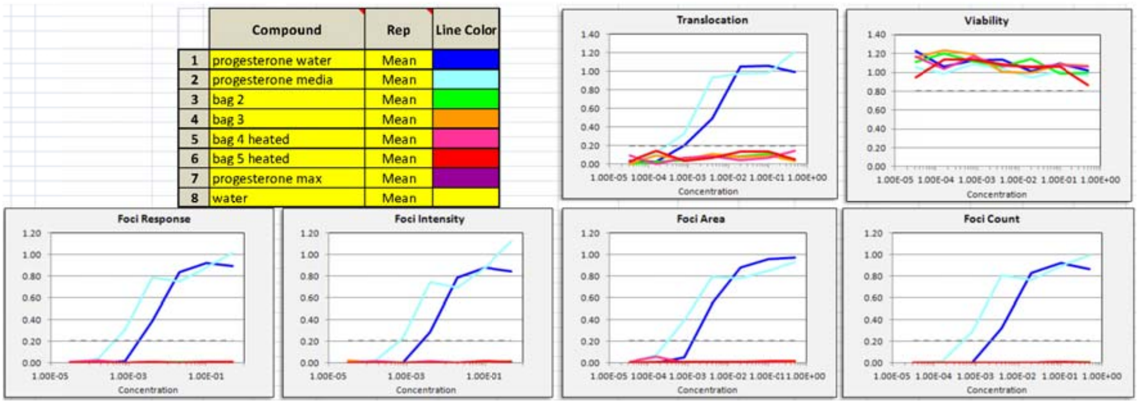

#

#

		ER<br>EPP Assay - Estrogen Receptor α			AR<br>EPP Assay - Androgen Receptor			PR<br>Progesterone Receptor Assay		
compound/sample	CAS #:	foci fomation	degradation	toxicity	foci formation	translocation	toxicity	foci formation	translocation	toxicity
17β-Estradiol	50-28-2	POS	POS	100 μM	POS	POS	100 μM	not tested	not tested	not tested
DHT (5α-Androstan-17β-ol-3-one)	521-18-6	POS	POS	*	POS	POS	*	not tested	not tested	not tested
Progesterone	57-83-0	NEG	NEG	100 μM	POS	POS	100 μM	POS	POS	*
B		NEG	NEG	*	NEG	NEG	*	NEG	NEG	*
LG		NEG	NEG	*	NEG	NEG	*	NEG	NEG	*
B+LG diluted		NEG	NEG	*	NEG	NEG	*	NEG	NEG	*
Bag 1		NEG	NEG	*	NEG	NEG	*	NEG	NEG	*
Bag 2		NEG	NEG	*	NEG	NEG	*	NEG	NEG	*
Bag3		NEG	NEG	*	NEG	NEG	*	NEG	NEG	*
Bag 4 (heated)		NEG	NEG	*	NEG	NEG	*	NEG	NEG	*
Bag 5 (heated)		NEG	NEG	*	NEG	NEG	*	NEG	NEG	*
				* > 100μM or 0.5x			* > 100μM or 0.5x			* > 100μM or 0.5x

#
